# Recent advances in managing vascular occlusions in the cardiac catheterization laboratory

**DOI:** 10.12688/f1000research.13271.1

**Published:** 2018-04-24

**Authors:** Athar M. Qureshi, Charles E. Mullins, Larry A. Latson

**Affiliations:** 1 CE Mullins Cardiac Catheterization Laboratories, The Lillie Frank Abercrombie Section, Texas Children’s Hospital of Cardiology, 6621 Fannin Street, Houston, TX 77030, USA; 2 Internal Medicine/Cardiology, Baylor St. Luke’s Medical Center, 6621 Fannin Street, West Tower, 19th Floor, MC 19345C, Houston, TX 77030, USA; 3 Joe DiMaggio Children's Hospital and Center for Adult Congenital Heart Disease, Memorial Healthcare System, 1005 Joe Dimaggio Drive Pediatric Heart Station Hollywood, FL 33021, USA

**Keywords:** Vascular Occlusions, thrombotic lesions, cardiac catheterization laboratory, recanalization procedures

## Abstract

Vascular occlusions continue to be a significant cause of morbidity and mortality. The management of vascular occlusions in patients is complex, requiring specialized expertise in the cardiac catheterization laboratory and from other disciplines. Knowledge of currently available tools at the operator’s disposal is important to optimize the success of these procedures. In this review, we discuss some of the recent advances in recanalization procedures of vascular occlusions and thrombotic lesions in the cardiac catheterization laboratory.

## Introduction

Vascular occlusions and thromboses remain a significant cause of morbidity and mortality in patients, even in the current era. In contrast to the treatment of stenotic lesions, catheter-based recanalization procedures of vascular occlusions are relatively infrequently performed, and this is because of the expertise required to perform these procedures. At experienced centers, transcatheter treatment of vascular occlusions has been shown to be technically feasible, safe, and effective in most cases. The general principles of techniques involved for recanalization of vessels in the cardiac catheterization laboratory remain the same with regard to imaging, optimizing the approach to the lesion, angioplasty, and stenting ^1– 4^. However, technological advances in equipment used for recanalization procedures confer the theoretical advantage of facilitating shorter procedure times and improving outcomes in what are frequently complicated and tedious procedures. In this review, we will focus on some of the newer equipment that facilitates crossing vascular occlusions in children and adults with or without congenital heart disease. We will also discuss newer systems designed to treat acute/subacute thrombotic (occlusive and non-occlusive) lesions. This article is not meant to be an exhaustive review of all the recent technology available; rather, it highlights some of the more recent and commonly used equipment that we and others have found useful and pertinent to our field.

## Sheath/catheter/guidewire technology

In order to transmit the most amount of force in a forward direction across the vascular occlusion, the angle of approach of the tip of the penetrating implement must be in as straight a line as possible with the center of the occlusion cap ^1– 3^. Self-fabricated guide and glide systems (guide catheter with co-axial hydrophilic catheter) and long sheaths (for example, Mullins sheaths, Cook Medical, Bloomington, IN, USA) remain useful in these situations. Newer guide catheters that are short do not need to be modified and can be connected to Y-shaped adapters in their current configuration through which glide catheters can be easily advanced. In taller patients or when the occluded lesion is distant from the access site, modification of guide catheters may still be necessary. Recently, steerable sheaths have become available and may shorten procedure times (particularly time spent to engage lesions that originate from challenging, acute angles). The Oscor Destino Twist (Oscor Inc., Palm Harbor, FL, USA) and the Agilis NXT Steerable Introducer (St Jude Medical, St Paul, MN, USA) are available in sizes of 6.5 to 13 French and 8.5 French, respectively. Both of these sheaths are steerable and available in many different curve options at the tip in addition to providing fixation of the curve. These sheaths, though expensive, may reduce procedure times during complex interventions ^5^, and we have found these sheaths to be very effective when trying to recanalize lesions that originate from acute angles (for example, the right lower pulmonary vein). It is important to remember that the vessel accessed for the treatment of vascular occlusions/thromboses is vital to help achieve the optimal angle of approach to the lesion. Unconventional vascular access (for example, percutaneous axillary artery ^6, 7^ and common carotid artery ^8– 10^ access) has now been adopted in the treatment of congenital heart disease. These access routes may facilitate the treatment of vascular occlusions/thromboses of major vessels originating from the abdominal aorta (for example, the superior mesenteric artery, celiac trunk, inferior mesenteric artery, and renal arteries). Access from more traditional approaches (for example, the femoral artery) may not be optimal and this is because of the acute angles of origin of these vessels.

Owing to the extensive need for recanalization of chronic total occlusions (CTOs) in the coronary arteries and lower extremities in the adult population, numerous guidewires specifically designed for crossing occluded lesions are now available, so complete review of these is beyond the scope of this review. In our recent experience, these CTO wires can be used in lieu of crossing occluded lesions with transeptal needles or radiofrequency perforation in many cases, although these modalities remain necessary in some instances.

**Figure 1.**
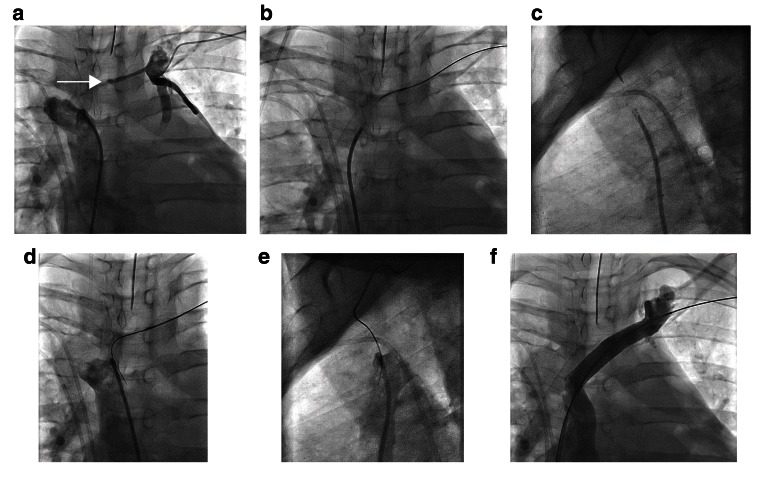
Owing to multiple central venous occlusions, a 36-year-old woman with chronic liver disease was deemed not to be a candidate for liver transplantation. ( **a**) An occluded left innominate vein (arrow) is seen. Initial attempts to cross the lesion with glide wires from a left basilic venous approach were unsuccessful. Via a shaped and modified catheter through a supporting sheath, the lesion was engaged with a Confianza wire using antero-posterior ( **b**) and lateral ( **c**) projections and simultaneous injections in the superior vena cava as a target. After the guidewire was incrementally advanced, angiograms in the superior vena cava confirmed intravascular position in the antero-posterior ( **d**) and lateral ( **e**) projections. The lesion was ballooned and eventually stented ( **f**), facilitating listing for liver transplantation.

From the perspective of inventory and stocking a cardiac catheterization laboratory, it is advisable to be familiar with a subset of CTO wires that can be used for a variety of procedures and are available to one’s facility. The Confianza wire (Abbot Vascular, Santa Clara, CA, USA), also known as “Conquest Pro” wire in some parts of the world, is a 0.014-inch guidewire that is available in tip loads of 9 and 12 grams. This guidewire is invaluable to most pediatric/congenital interventional cardiologists, as we and others have used this guidewire for the perforation of atretic pulmonary valves ^11^, among other indications. The distal end of the tip is uncoated to facilitate penetration of the lesion, and the rest of the tip is hydrophilic to facilitate movement once the lesion is crossed ( Figure 1). It may also be advantageous to stock guidewires that are available in both 0.014- and 0.018-inch calibers (for example, Victory guidewires, Boston Scientific, Natick, MA, USA). These guidewires are specifically made to cross resistant lesions and are available in tip loads of 12, 18, 25, and 30 grams. The tip consists of a polymer sleeve that has hydrophilic coating, and the core of the wire is stainless steel, which allows better transmission of force and good support. The larger-caliber, higher-tip load wires are helpful when crossing larger-diameter vessels that have been chronically occluded (for example, the superior vena cava).

In many instances, despite adequate support using sheaths and guide catheters, the penetrating guidewire will be able to be advanced only a short distance. In these instances, advancing a micro-catheter over the guidewire can serve a number of purposes. First, by advancing the micro-catheter in increments, enough support is provided for the guidewire to be advanced far enough either to allow snaring from the other end if accessible or to allow a balloon to track (by facilitating the part of the wire that has more support to be across the target lesion). Second, once the micro-catheter is distal enough, the wire may be exchanged for a guidewire with more support if needed ^12^. Finally, a micro-catheter that is available with angles at the tip provides access to vessels that come off angles that otherwise would be challenging to enter with a guidewire alone. The Echelon 14 Micro Catheter (Medtronic, Minneapolis, MN, USA) is one such micro-catheter that is available in tips that are straight as well as at 45 and 90 degree angles.

In some cases, it may be possible to enter via only a subintimal approach with intentional creation of a plane of dissection ^13^. Withdrawing the guidewire and using a different CTO wire (or the same wire) that is shaped at the end can enable re-entry into the true lumen. A number of devices now available are specifically designed to re-enter the true lumen, and operators may benefit from having at least one system they are familiar with. Subintimal angioplasty has been shown to be a safe and effective way of restoring vessel patency ^14^, and this has been our experience as well. A variety of crossing devices that can help traverse vascular occlusions are also available. They may facilitate greater technical success rates as a primary modality of crossing as opposed to using guidewires initially ^15^. Intravascular ultrasound (IVUS) has been shown to be helpful in guiding endovascular stenting for iliac arterial disease ^16^, and IVUS and other imaging modalities can be used to complement angiography to facilitate treatment of vascular occlusions if needed.

## Thrombectomy and thrombolysis systems

Over the years, a variety of thrombectomy systems have been used to treat vascular thrombosis. Rheolytic systems, though effective, can result in hemodynamic instability due to adenosine-mediated release mechanisms, and prior-generation aspiration systems required manual aspiration ^4^. Thrombolysis with lytic therapy can result in significant bleeding complications in some patients because of the dose and duration of lytics required. The thrombectomy and thrombolysis systems we discuss below overcome some of these drawbacks of prior generations of devices.

### Penumbra/Indigo System

**Figure 2.**
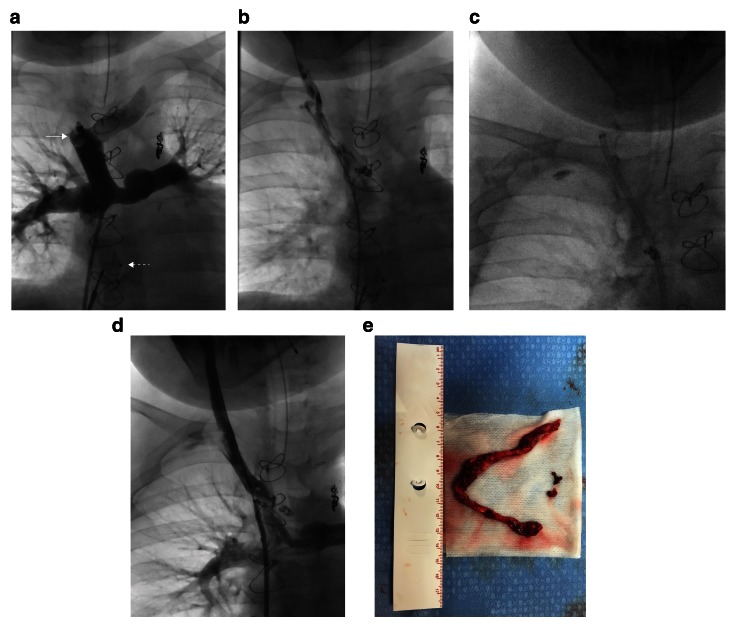
A 9-year-old boy with a Fontan circulation presented with unilateral right-sided facial and neck swelling due to acute thrombus formation in the right innominate vein and right internal jugular vein. ( **a**) An occlusive clot can be seen at the right innominate vein–superior vena cava junction (solid arrow). The dotted arrow points to a device that was temporarily placed in the fenestration to minimize the potential risk of systemic embolization. Once the lesions were crossed, an angiogram demonstrates numerous filling defects along the entire length of the right internal jugular vein and right innominate vein ( **b**). After the Penumbra/Indigo system ( **c**) was used in conjunction with balloon angioplasty, resolution of the thrombus with restoration of normal flow occurred ( **d**). Wash-in from the right subclavian vein is also seen. Large amounts of thrombi were extracted by using the Penumbra/Indigo system ( **e**), and facial swelling resolved.

The Penumbra/Indigo system (Penumbra, Alameda, CA, USA) was initially designed for use in the cerebrovascular arterial system and subsequently has been optimized for use in the central and peripheral vascular system ^17– 19^ ( Figure 2). Compared with manual aspiration and wall suction systems, the Penumbra/Indigo systems provide increased flow rates and more suction by using a machine-assisted vacuum technique. The catheters are available in 3, 5, 6, and 8 French sizes and track easy over guidewires. The catheter is advanced until flow ceases, indicating that the clot has been engaged. With the system turned on, the appearance of free-flowing blood indicates that the thrombus has been aspirated/cleared. A separator wire can also be used within the catheter to prevent the tip/catheter from clotting by in-and-out manipulation. The catheter may also be passed distally and proximally to extract small clots. In smaller patients, we aim to maintain euvolumia by transfusing the same amount of blood that is seen in the aspirate. In a recent trial, the Penumbra/Indigo system was evaluated in 85 patients with acute ischemia, incomplete resolution of thrombi with other procedures, or distal emboli as a result of other procedures. The investigators reported efficacy and safety using the Penumbra/Indigo system when used alone or as an adjunctive therapy ^19^.

### The AngioVAc System

The AngioVAc System (AngioDynamics, Latham, NY, USA) uses the concept of extracorporeal veno-venous bypass to extract large amounts of thrombi/vegetation. The drainage cannula is a specially designed 22 French cannula with a funnel configuration at its end that is advanced close to the area of large thrombi. This is connected to a filter and centrifugal pump that returns filtered blood to the body via another large cannula that is placed in a different large central vein. The entire procedure can be performed percutaneously. When the clot burden is extensive or thrombi are in large chambers (for example, the right atrium), this system can be very effective and can avoid the need for open-heart surgical extraction ^20, 21^. Although we and others have used this system in patients with congenital heart disease ^22, 23^, the size of the cannula currently limits this application to larger patients.

### EKOS Endovascular System

**Figure 3.**
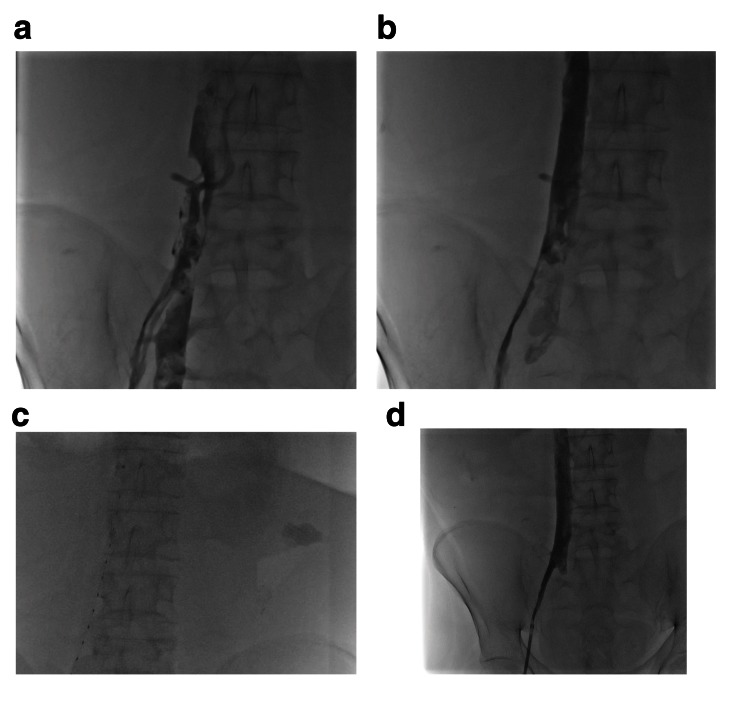
A 27-year-old male with short gut syndrome and a history of multiple abdominal surgeries. ( **a**) The patient developed multiple thrombi in his right external iliac vein, right common iliac vein, and inferior vena cava. ( **b**) After mechanical thrombectomy, multiple filling defects remained. An EKOS Endovascular System catheter was placed ( **c**) and low-dose tissue plasminogen activator was administered, resulting in complete resolution of the thrombi ( **d**).

The main principle of the EkoSonic Endovascular System (EKOS Endovascular System, EKOS Corporation, Bothell, WA, USA) is to use sonic energy to enhance thrombolysis/fibrinolysis. This process is a result of better lytic distribution and penetration in the thrombus ( Figure 3). As a result, the dose/infusion time of thrombolytic agents is decreased, as are the bleeding complications associated with thrombolytic therapy ^24^. The EKOS Endovascular System consists of a 5.4 French infusion catheter (compatible with a 6 French sheath) with multiple side holes through which lytic therapy is dispersed. The treatment zone is highlighted by radiopaque markers on the catheter, which are available in varying lengths that correspond to treatment ranges from 6 to 50 cm. Once the catheter is in place, an ultrasound core is advanced through this catheter. The ultrasound core is connected to a console that controls the ultrasonic waves and lytic therapy is administered continuously for 24 hours (depending on the situation, less or more time may be required). In a recent prospective study for the treatment of pulmonary emboli in adults ^25^, the EKOS Endovascular System was found to be effective in reducing thrombus burden and secondary markers of pulmonary emboli (for example, right ventricular dilation and pulmonary artery pressures). Importantly, no patient experienced an intracranial bleed. There is limited experience of the use of the EKOS Endovascular System catheter system in children with congenital heart disease; however, in our experience ^4, 26^, results thus far have mirrored what has been reported in adults.

## Future directions

There have been many advances in recent years in the treatment of vascular occlusions and thromboses, and it is likely that further refinement of these techniques and equipment will occur in the future. Miniaturization of the tools used in these procedures will expand their use to smaller children. Collaboration among interventional cardiologists, interventional radiologists, vascular surgeons, and other specialists will be essential to refine these techniques.
